# Cancer of Pylorus

**Published:** 1868

**Authors:** George P. Hannawalt

**Affiliations:** Washington, D. C.


### Cancer of Pylorus. By George P. Hannawalt, M. D., Washington, D. C.

About 20th of August last I was called to see Mr. B——, a resident of the city of Washington, brewer, and a native of Germany. He stated that he had been suffering for several months, chiefly from pain in the stomach and right side, with occasional nausea and vomiting. He also complained of obstinate constipation of the bowels and acid eructations. His appearance was that of a large, well-formed man, about thirty-eight years of age, somewhat emaciated, and with a dull, weary and cachectic expression of countenance.

A careful physical examination revealed a hard, somewhat moveable tumor in the inferior portion of the epigastric and the right hypochondriac regions. It was not very sensitive to the touch, as considerable pressure could be made upon it without causing pain.

These symptoms, together with the patient's statement that his mother had died of "tumor of the womb," led to the following diagnosis: Cancerous growth of ihe stomach or liver—possibly both involved.

Purgative medicines were ordered, which, after three days, produced copious pale-colored stools. Full doses of bismuth and morphia were given to correct the acidity of the stomach and allay pain. The bowels were kept open by saline cathartics. Two weeks subsequently jaundice ensued—the conjunctiva and entire surface of the body being deeply colored, the urine loaded with bile, and the exhalations from the body characterized by the peculiar icteric odor.

The patient took food regularly, but described his appetite as "gnawing"—by no means good. At this time the most distressing symptom was the vomiting, which usually occurred within an hour after eating, consisting of the food taken, and a dark coffee-colored fluid of a sickening sourish smell. The pain was not constant, but severe, and, as described by the patient, "shooting frum the pit of the stomach to both sides of the body."

These distressing symptoms gradually increased, despite the varied treatment resorted to—the patient's only relief being in the stupor produced by opiates. On October 18th, death from exhaustion released the sufferer.

Autopsy; twelve hours after death. Slight rigor mortis. The stomach was enormously extended, being at least three times its normal size, the greater curvature extending far below the umbilicus—containing about two quarts of brownish fluid, in which were numberless small black scales or clots. A scirrhous tumor, the size of a very large lemon, was found in the situation before-mentioned, involving the pylorus, part of the duodenum, orifice of the gall-bladder and biliary ducts. The pyloric orifice was so much obstructed as to admit the end of the little finger with great difficulty, while repeated efforts to introduce a small silver probe through either of the biliary ducts was unsuccessful.

The gall-bladder was filled with white, tenacious fluid, of the conistence of thick cream, together with more than fifty calculi, four of which were the size of large hazel nuts, the remaining ones being quite small. These larger concretions were of a reddish-yellow color, except a central portion or nucleus, which, in color and consistence, appeared carbonaceous. The smaller ones seemed to consist, wholly of the same material as the nuclei of the larger ones. The mucous coat of the sac was perforated by very small ulcers; in the upper part, the membrane hung in disintegrated shreds.

The liver, throughout, was firmer in texture and of a darker color than normal, and, in the region of the tumor, was indurated and closely adherent to the diaphragm.

A close examination failed to discover any further lesion in the thorax or abdomen.

[No more appropriate place than this offers itself to record the autopsies of two cases of cancer, recently coming under our knowledge. The first was that of an Irishman about sixty years of age, under observation but a few days, and supposed to have cancer of the stomach, though the symptoms were obscure. Very little pain was complained of, and physical signs, save emaciation, were disguised, and considerable abdominal enlargement, from ascites. Two days before death severe hematemesis occurred, rendering the idea of cancer of the stomach plausible, and the patient sank rapidly. At the autopsy, the stomach, esophagus and intestines were healthy. The stomach and intestines contained a quantity of dark blood, becoming quite black a short distance from the pyloric orifice, no doubt from the action of the gastric and intestinal fluids. No possible source of hemorrhage was discovered, rendering it certain that the cause was the passive congestion common in such cases. The liver, anteriorly and superiorly, presented the appearance of cirrhosis; from the under surface of the right lobe, near its junction with the lobulus caudatus, grew a yellowish-white tumor, as large as a medium-sized orange. The diseased condition gradually shaded off into the hepatic tissue. The tumor could not have been detected by palpation; it was a soft brain-like mass, and microscopic examination proved it to be undoubtedly encephaloid cancer. The gall-bladder was somewhat distended and contained a large number of gall-stones, consisting principally of inspissated bile. Several pints of serum were found in the peritoneal cavity. No disease existed elsewhere.

The second case was under the care of Dr. Soule, at the City and County Hospital, and was diagnosticated cancer of the pyloric orifice of the stomach. Considerable emaciation existed. There was not much pain, but for some time previous to death all food was ejected shortly after eating. Post-mortem examination showed the stomach and its orifices to be perfectly healthy. Within the duodenum, however, perhaps four inches from the pyloric orifice, was a large encephaloid cancer, occupying an area of ten or twelve inches, and so softened as to be partially washed away by the water used to cleanse it. At this point, the caliber of the intestine was greatly encroached upon.

Through the center of the mass passed the common bile-duct, the exit of the bile being thus impeded. The gall bladder was greatly distended, and contained several large biliary concretions. Examination of other organs was not made.]—*H. G., Jr., Pacific Medical and Surgical Journal.*

